# Determination of Intracellular Vitrification Temperatures for Unicellular Micro Organisms under Conditions Relevant for Cryopreservation

**DOI:** 10.1371/journal.pone.0152939

**Published:** 2016-04-07

**Authors:** Fernanda Fonseca, Julie Meneghel, Stéphanie Cenard, Stéphanie Passot, G. John Morris

**Affiliations:** 1 UMR GMPA, AgroParisTech, INRA, Université Paris-Saclay, Thiverval-Grignon, France; 2 Asymptote Ltd, St John’s Innovation Centre, Cambridge CB4 0WS, United Kingdom; University of California at Berkeley, UNITED STATES

## Abstract

During cryopreservation ice nucleation and crystal growth may occur within cells or the intracellular compartment may vitrify. Whilst previous literature describes intracellular vitrification in a qualitative manner, here we measure the intracellular vitrification temperature of bacteria and yeasts under conditions relevant to cryopreservation, including the addition of high levels of permeating and nonpermeating additives and the application of rapid rates of cooling. The effects of growth conditions that are known to modify cellular freezing resistance on the intracellular vitrification temperature are also examined. For bacteria a plot of the activity on thawing against intracellular glass transition of the maximally freeze-concentrated matrix (Tg’) shows that cells with the lowest value of intracellular Tg’ survive the freezing process better than cells with a higher intracellular Tg’. This paper demonstrates the role of the physical state of the intracellular environment in determining the response of microbial cells to preservation and could be a powerful tool to be manipulated to allow the optimization of methods for the preservation of microorganisms.

## Introduction

In order to develop better methods of cryopreservation for microbial cells it is necessary to understand the cellular response to the stresses encountered during freezing and thawing. The widely accepted model of freezing injury to cells is the “two factor hypothesis”[1] which has largely been developed with mammalian cells and in many cases there is good correlation between survival on thawing, visualization of intracellular ice formation and modeling of osmotic dehydration during cooling. This hypothesis applies to the yeast *Saccharomyces cerevisiae* [2,3], but does not necessarily hold true for prokaryotes [4]. The two factor hypothesis defines conditions under which intracellular ice formation occurs, but is silent on the physical state of the intracellular compartment in highly shrunken cells in which intracellular ice is absent. Recently, intracellular vitrification of mammalian oocytes, achieved by high solute concentration and rapid cooling, and the deleterious effects of a relatively slow warming rate, which results in devitrification and intracellular ice formation during warming have been examined [5].

It is now recognized that the intracellular compartment is an aqueous environment which is extremely crowded [6]. Osmotic removal of water from the cytoplasm leads to a buildup in the concentration of intracellular solutes and an increase in the packing density of macromolecules. In osmotically stressed eukaryotic cells the formation of an intracellular glass has been demonstrated [6,7]. The intracellular environment of prokaryotes is more packed than eukaryotes, the macromolecular concentrations in *E*.*coli* in isotonic cells are reported to be 200 g.L^-1^ protein, 75 g.L^-1^ RNA and 10–20 g.L^-1^ DNA compared with a total macromolecular concentration of 50–100 g.L^-1^ for human brain cells [8]. Following osmotic stress in bacteria the diffusion rates for intracellular molecules decrease [9,10], the macromolecule concentration may exceed 400 g.L^-1^ [8] leading to a glassy state in the cytoplasm [11]. This glass has the properties of a dense suspension of colloidal particles (colloid glass) rather than that of a molecular glass. A colloid glass behaves like a molecular sieve [9] allowing the free passage of small molecules whilst restricting the diffusion of bigger ones.

During freezing in the environment ice forms externally to the cells which become compartmentalized into zones of freeze concentrated liquid and dehydrate as the external solution withdraws water from the cell. Cells are thus simultaneously exposed to two stresses; an increase in external osmolality and a decrease in temperature. During extracellular freezing, unicellular microorganisms undergo an intracellular glass transition, as detected by differential scanning calorimetry, at high subzero temperatures (between -10°C and −26°C) [12]. In contrast to intracellular freezing, vitrification does not result in death and cells may survive low temperatures when warmed. The high internal viscosity following vitrification means that diffusion of oxygen and metabolites is slowed to such an extent that cellular metabolism ceases and the stresses associated with hypertonic solutions within the cell are removed. It has been argued [12] that vitrification will provide a survival strategy and will determine the low temperature limit for growth for bacteria. In the current study we examine the intracellular vitrification of bacteria and yeasts under conditions relevant to cryopreservation, including the addition of high levels of permeating and nonpermeating additives and the application of rapid rates of cooling. The effects of growth conditions that are known to modify cellular freezing resistance on the intracellular vitrification temperature are also examined. The role of the physical state of the intracellular environment in determining the response of cells to the stresses encountered during cryopreservation is discussed.

## Materials and Methods

**Fig 1:**
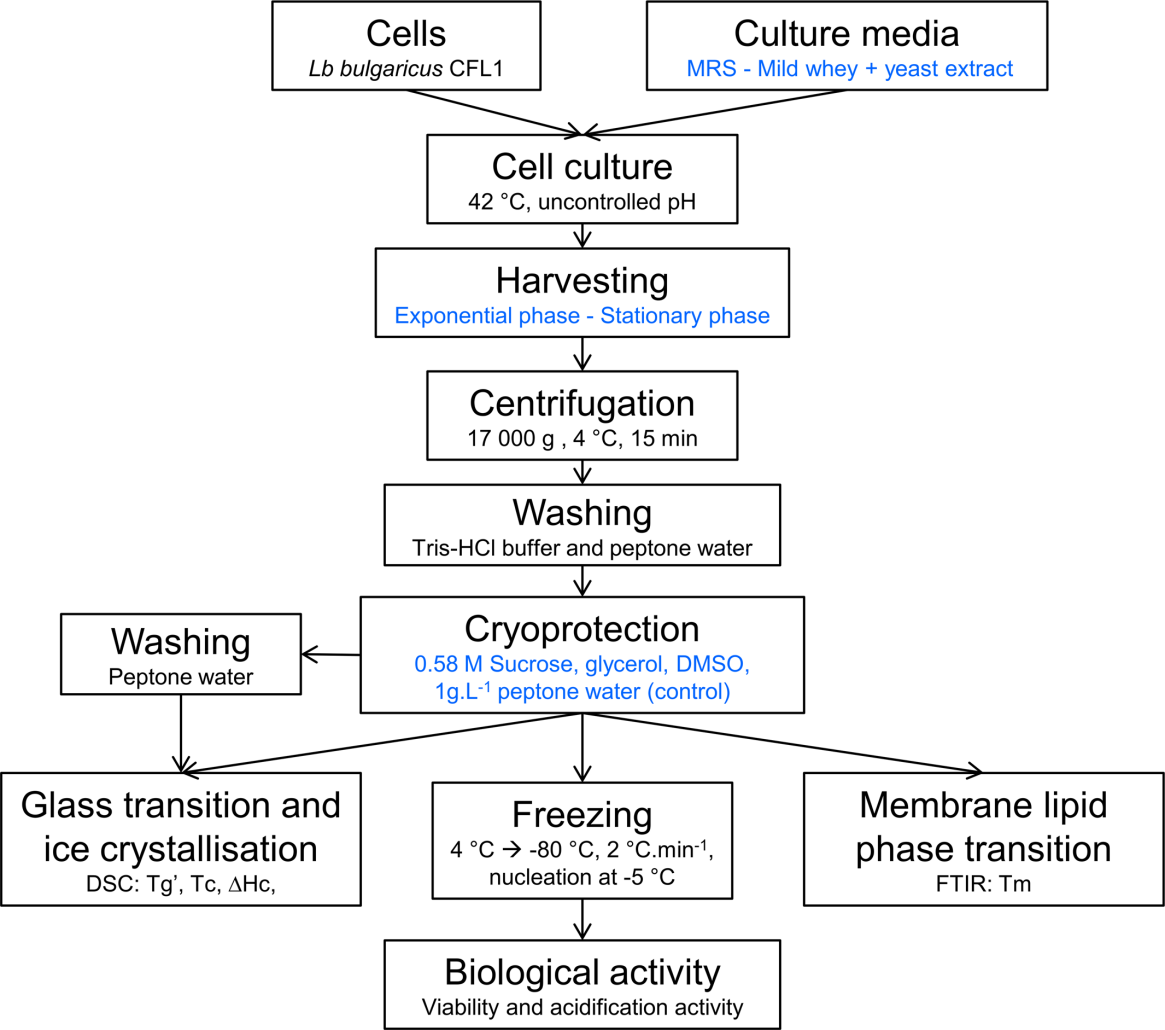
Diagram of the experimental procedure used and the analysis performed on *L*. *bulgaricus* CFL1 samples. DSC, FITR, viability and acidification activity measurements were performed on at least three independent biological replicates. Steps involving different conditions are in blue. Similar experimental procedure was used for *S*. *cervisiae* 338.

The experimental approach used during this study and the main parameters investigated are summarized in Fig 1.

### Cells

*Lactobacillus delbrueckii* ssp. *bulgaricus* CFL1 was obtained from CIRM -BIA (International Center of Microbial Resources, Rennes, France) and *Saccharomyces cerevisiae* 338 was provided by CIRM-Levures (International Center of Microbial Resources, Thiverval-Grignon, France). Inocula were stored at −80°C and were thawed at growth temperature for 5 minutes before inoculation.

### Culture media

For *L*. *bulgaricus* CFL1 two different culture media were used: de Man–Rogosa–Sharpe (MRS) broth (Biokar Diagnostics, Beauvais, France) and mild whey-based medium. MRS broth was prepared following the supplier’s instructions and it was sterilized at 121°C for 15 min. The mild whey medium was prepared by suspending 60 g of mild whey powder from Euroserum (Port-sur-Saône, France) in one litre of demineralized water and was heated at 110°C for 20 min. The supernatant obtained after centrifugation (17 000 g for 30 min at 4°C) and filtration (330 mm) was supplemented with yeast extract at a concentration of 5 g.L^-1^ (Organotechnie SAS, La Courneuve, France).The mild whey medium was then sterilized at 110°C for 20 min.

For *S*. *cerevisiae* 338, Yeast extract-Peptone-Dextrose (YPD) medium was prepared from elementary ingredients (10 g.L^-1^ yeast extract, 10 g.L^-1^ bactopeptone, 10 g.L^-1^ glucose) and sterilized at 121°C for 15 min.

### Cell culture

Bacterial cultures were maintained at 42°C. Two different growth phases (determined by OD measurements) were studied: the end of the exponential phase and the stationary phase (harvest 3 hours after the end of the exponential phase).

Yeasts were cultured out at 25°C and harvested in exponential phase (18 h culture).

Bacteria and yeast cells were harvested by centrifugation at 17000 g for 15 min at 4°C. Cells grown in mild whey based broth were subjected to two Tris buffer washes prior to being washed in peptone water to ensure the removal of residues of whey protein. Then, cells were washed twice with peptone water (1 g.L^-1^).

### Physical treatments prior to freezing

Cells were exposed to osmotic stress conditions by the addition of solutions of cryoprotective additives (0.5 h incubation time at 25°C). Cell pellets were analyzed by differential scanning calorimetry (DSC) after cryoprotection and centrifugation and following either one or 2 washes in peptone water (1 g.L^-1^).

Cell pellets obtained after several washes and centrifugation (13 000 g for 5 min at 4°C) were used as controls.

### Cryoprotective solutions

Three molecules were added to *L*. *bulgaricus* at the same molar concentration (0.58 M): glycerol 5.2% w/v (Sigma-Aldrich, France), sucrose 20% w/v (VWR Chemicals, France) and dimethyl sulfoxide, DMSO 4.5% w/v (VWR Chemicals, France), corresponding to molar concentrations currently employed for the production of starter cultures. Each aqueous solution was prepared in saline water (NaCl 0.15 M) with 0.2% of Snowmax (in order to facilitate ice nucleation) and sterilized at 121°C for 15 min.

### Determination of glass transition temperature and ice crystallization by DSC

The intracellular glass transition temperature (Tg) was determined by differential scanning calorimetry as described previously [12]. The glass transition temperature measured by DSC is Tg', that is the Tg of the maximally freeze-concentrated matrix. About 10–50 mg of each cell pellet was scanned following cooling at 2, 10 and 50°C.min^-1^ to -100°C and -150°C depending on the sample (lowest temperatures for samples containing glycerol and DMSO) and heating to 20°C at 10°C min^-1^. In some experiments samples were examined during multiple freeze thaw cycles in the DSC. Aqueous solutions of protective molecules were also analysed for comparison to protected cells. They generally exhibit two thermal events characterizing the glass transition. They represent the glass transition temperature of the maximally freeze concentrated phase (the lower value, Tg’) [13,14] and the softening temperature at which the system exhibits an observable deformation (viscous flow in real time) under its own weight (the higher value) [15]. Ice crystallization was also characterized by the latent heat of ice crystallization (ΔHc, in J.g^-1^) from the area of the exothermic peak and the extrapolated peak onset temperature of ice crystallization (Tc).

### Determination of intracellular ice formation by CryoSEM

The intracellular ice formation of *S*. *cerevisiae* cells was investigated by applying cooling rates from 2 to 50°C min^-1^. Cell suspensions were frozen to -100°C in 0.5 mL straws (IMV Technology, L’Aigle, France) and transferred to liquid nitrogen. A controlled rate freezer (EF600-103, Asymptote, Cambridge, UK) was used for slow cooling rates. Direct transfer of straws to molecular sieves (3.2 mm) equilibrated with liquid nitrogen were used to achieve a rate of 50°C min^-1^ within the straw [16]. Cell suspensions were examined in the frozen state using cryo scanning electron microscopy (CryoSEM), as described previously [4].

### Determination of membrane lipid phase transition temperatures

Membrane lipids are typically found in a liquid crystalline phase under physiological conditions and undergo a phase transition to a gel state upon cooling and vice versa upon warming. Such transitions were monitored by Fourier Transformed Infra Red Spectroscopy (FTIR) and the corresponding lipid membrane transition temperatures (Tm) were determined as described previously [17]. Cell pellet containing cryoprotective additive was sandwiched between two CaF_2_ windows and scanned following cooling to -50°C and heating to 80°C at 2°C.min^-1^.

### Freezing and thawing protocol

A controlled rate freezer (EF600-103, Asymptote, Cambridge, UK) modified as described previously [18] by the addition of a module made of aluminum was used in this work. The module was fixed to the flat cooling plate of the EF600-103. Thermocouples (K type) were used to measure the sample temperature inside the vials (two) and the plate temperature (two). The thermocouple was connected to a computer to monitor the sample temperature.

The sample plate of the freezer was precooled at 4°C. Vials containing 1ml aliquots were placed in the module and equilibrated during 15 minutes before starting the cooling profile.

The EF600-103 was programmed to cool at 5°C min^-1^ from 4°C to -5°C, hold this temperature for 15 minutes to allow manual nucleation, and then cooled at 2°C min^-1^ from -5°C to -80°C. The samples were held in the EF600-103 at -80°C for 1 h after the cooling cycle was complete, before being transferred to a -80°C freezer for 7 days.

The frozen cells were rapidly warmed during 120 s in a 42°C water bath until all the ice had melted before analysis (viability and acidification activity).

### Loss of viability on thawing

Viability of bacterial suspensions before and after freezing was assessed by the agar plate count method (MRS-agar), and the cell count was expressed in colony forming units per milliliter (CFU.mL^-1^).

### Loss of acidification activity on thawing

The quality of lactic acid bacteria is generally defined by both the replicating capacity of cells in culture and their ability to acidify a certain medium. The CinAc system (AMS, Frépillon, France) was used to measure the acidification activity of *L*. *bulgaricus* suspensions [19]. This automatic real time pH measurement is more meaningful and reproducible than the enumeration of colony forming units on agar. Because most lactic acid bacteria form chains of varying length that differ according to the stage of the production process [20], colony formation give less reliable results.

Acidification was measured in triplicate at 42°C using reconstituted skim milk (100 g.L^−1^ skim milk powder) (EPI Ingredients, Ancenis, France), heat-treated at 110°C for 15 min in 150 mL flasks, and stored at 4°C for 24 h before use. The flasks were inoculated with 100 μL of bacterial suspension. The pH was continuously measured by the CinAc system and led to the determination of the time necessary to reach the maximum acidifying rate in milk (tm, in minutes).The descriptor tm was used to characterize the acidification activity of bacterial suspensions. The higher the tm value was, the longer the latency phase was and the lower the acidification activity. Moreover, the specific acidification activity tspe (in min.log (CFU.mL^−1^)^−1^) was defined as the ratio of tm to the corresponding log of the cell concentration [21]. The descriptor tspe makes possible the quantification of the biological activity of *L*. *bulgaricus*, including the physiological state and viability. By quantifying tspe before and after freezing we were able to determine the loss of specific acidification activity during freezing: tspe after freezing minus tspe before freezing (in min.log (CFU.mL^−1^)^−1^).

### Statistical analysis

Two sample permutation tests (Wilcoxon-test, two-sided, normal approximation with continuity correction, R3.2 software) were conducted to contrast data concerning DSC, FTIR and loss of acidification activity.

## Results

### Freezing and thawing of *Saccharomyces cerevisiae*

**Fig 2:**
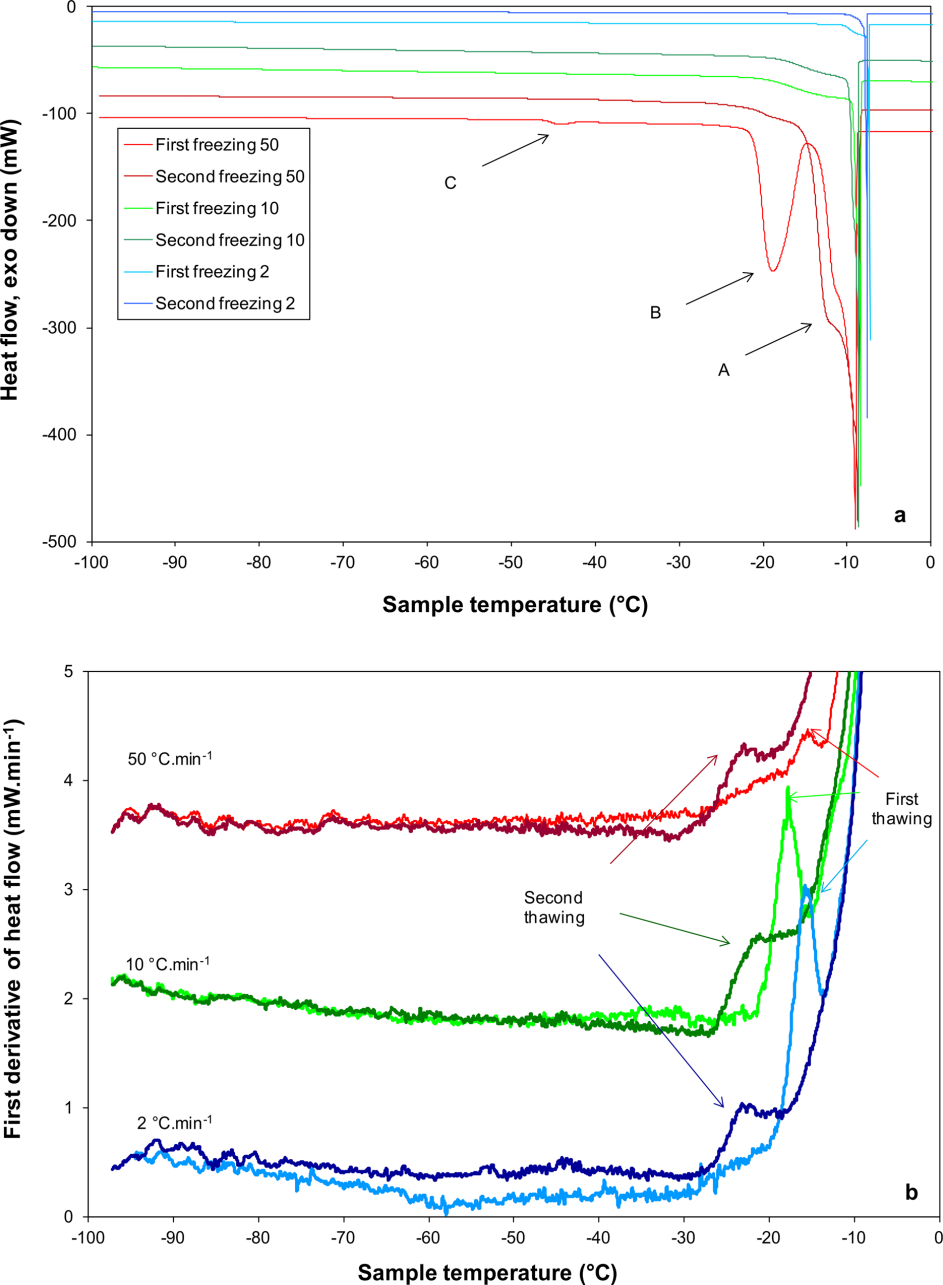
DSC traces of *S*. *cerevisiae* 338 cells during cooling. (a) DSC heat flow traces at 50°C.min^-1^ (red curves), 10°C.min^-1^ (green curves) and 2°C.min^-1^ (blue curves) and (b) DSC first derivative of the heat flow during warming at 10°C.min^-1^. In all figures the effects of a repeat freeze thaw were also determined. Capital letters A, B and C in Fig 2A correspond to exothermic events indicated in Table 1.

DSC scans of *S*. *cerevisiae* during cooling at 50°C.min^-1^, 10°C.min^-1^ and 2°C.min^-1^ (Fig 2A) and subsequent warming at 10°C.min^-1^ (Fig 2B) reveal a number of transitions, which are summarized in Table 1. For all treatments the effects of a repeated freeze thaw cycle are also presented. The high temperature exothermic event (Tc: -6°C to -10°C, marked A in Fig 2A) observed at all cooling rates corresponds to the spontaneous freezing of the aqueous medium in which the cells were suspended. During initial cooling at 50°C.min^-1^ a major peak is detected at -17°C (marked B in Fig 2A), with a smaller peak consistently observed at -43°C (marked C in Fig 2A). These two latter peaks are not seen when the cells are thawed and cooled again at 50°C.min^-1^. At rates of cooling of 10°C min^-1^ and lower, the minor peak at -43°C is sometimes observed, but the major peak at -17°C is absent. From enthalpy measurements (Table 1), more ice forms at slow rates of cooling, following cooling at 50°C min^-1^ the total ice formation is 200 ΔHc J g^-1^, which is 92% of the value observed at 2°C.min^-1^. Following a second freeze at 50°C.min^-1^ more ice is observed to form (an additional 2%) but the difference is not significant (*p* < 0.05) between treatments.

**Table 1:** Summary of the thermal events observed by DSC with *S*. *cerevisiae* 338 following freezing at different cooling rates.

Cooling rate (°C.min^-1^)	Initial freeze			First thaw	Repeat freeze			Second thaw
	Peak	Tc (°C)	ΔHc (J.g^-1^)	Tg’ (°C)	Peak	Tc (°C)	ΔHc (J.g^-1^)	Tg’ (°C)
50	A	-10.7 ± 3.5	-115 ± 9	-17.3 ± 1.3^a^	A	-11.1 ± 4.1	-204 ± 12	-21.8 ± 0.8^b^
	B	-16.7 ± 3.5	-84 ± 10					
	C	-42.6 ± 0.7	-1.1 ± 0.7					
10	A	-7.2 ± 1.0	-223 ± 20	-16.2 ± 1.0^a^	A	-7.2 ± 1.4	-223 ± 24	-19.7 ± 1.4^b^
2	A	-6.5 ± 0.7	-218 ± 8	-16.8 ± 1.4^a^	A	6.3 ± 0.9	-222 ± 15	-20.7 ± 2.1^b^

Tc: crystallization temperature in °C

ΔHc: latent heat of crystallization in J.g^-1^

Tg’: glass transition temperature in °C

The high temperature exotherm observed during cooling (A). corresponds to the freezing of the aqueous medium in which the cells were suspended and is observed in cell-free samples. Other exothermic events are indicated B and C

Means (of at least 5 measurements) are presented ± experimental standard deviations.

Superscripts letters (a and b) represent statistical contrasts between samples at the 95% confidence level.

At all rates of cooling examined, an intracellular glass transition is detected during warming at -16°C to -17°C from the peak of the first derivative of the heat flow (Fig 2B), this is associated with a change in heat capacity as the cells resume molecular mobility due to the decrease of viscosity at Tg’_._ There were no significant differences (p < 0.05) in Tg’ between the different cooling rates (Table 1). Following a second freeze, the intracellular Tg’ occurred at a lower temperature (-20°C to -22°C) with no significant differences among different cooling rates (*p* < 0.05). But there are significant differences between Tg’ following an initial freeze and Tg’ in cells which had been thawed and then refrozen.

**Fig 3:**
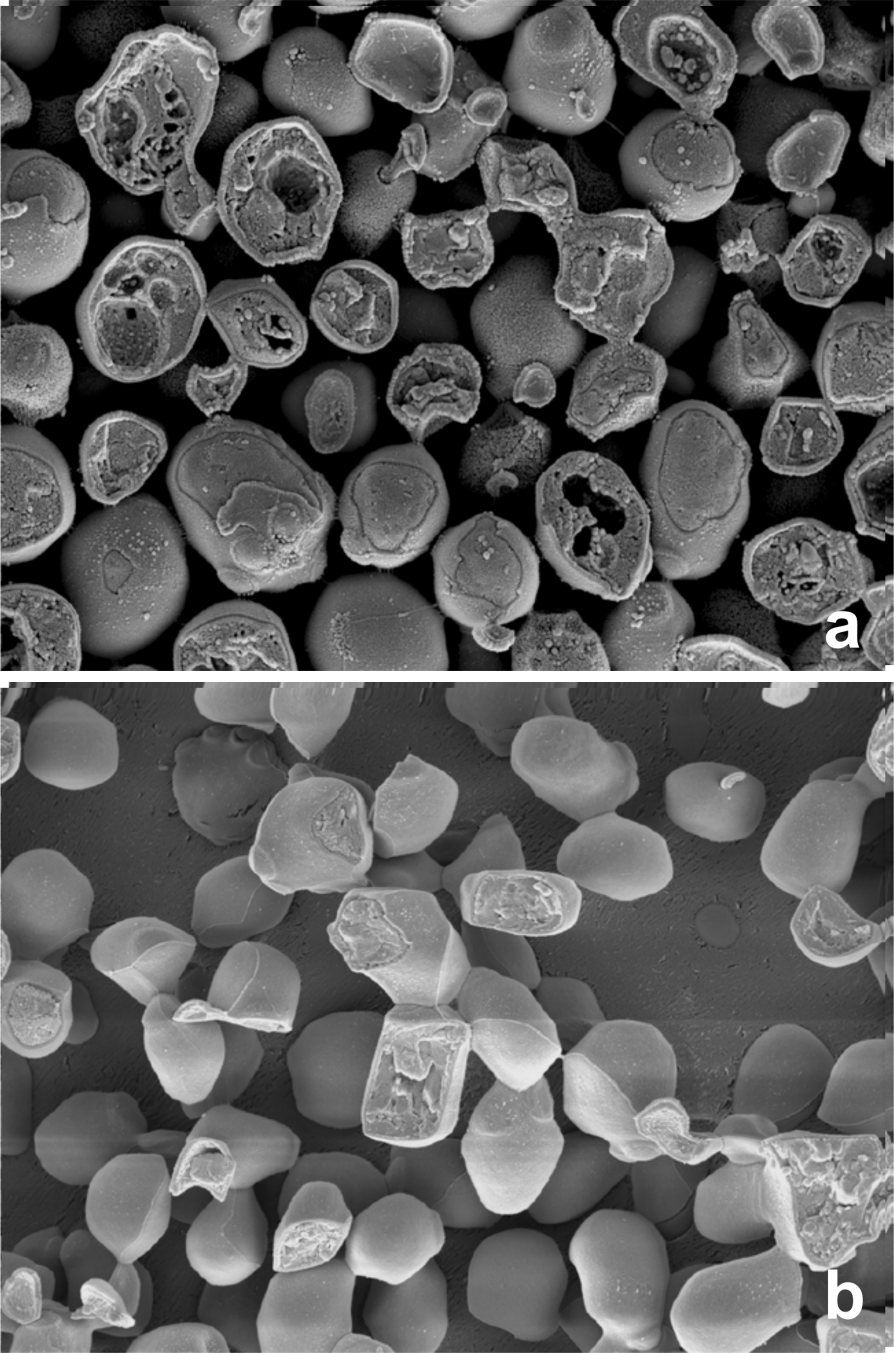
CryoSEM of *S*. *cerevisiae* 338 cells following cooling. (a) At 50°C.min^-1^ and (b) at 2°C.min^-1^. Note the large voids in cells cooled at 50°C.min^1^, caused by the formation of intracellular ice.

CryoSEM of *S*. *cerevisiae* 338 was carried out following cooling at 50°C.min^-1^, 10°C.min^-1^ and 2°C.min^-1^ (Fig 3). At 50°C.min^-1^ intracellular ice is obvious within fractured cells as large voids (Fig 3A), which is consistent with the exothermic event observed by DSC at this rate of cooling (Fig 2A, Table 1). At slower rates of cooling intracellular ice is not apparent (Fig 3B); cells appear non spherical and shrunken when compared with those in 3a and little structure is obvious within the cytoplasmic compartment of fractured cells, importantly there are no voids, indicative of intracellular ice within the cytoplasm (Fig 3B).

### Freezing and thawing of *Lactobacillus bulgaricus*

Similar experiments were carried out with *L*. *bulgaricus* CFL1 (Fig 1) under the same conditions as with *S*. *cerevisiae* reported above. At the rates of cooling examined a high temperature exotherm corresponding to the spontaneous freezing of culture medium was observed, but no other exotherms were observed during cooling (data not shown).

During warming, an intracellular Tg’ was observed and the values of intracellular Tg’ were dependent on the growth medium and the phase of culture (Table 2). In the case of the control sample (cells but no additives), no significant differences were observed according to the growth medium, but in the case of MRS growth medium, Tg’ was observed at a slightly lower temperature with cells from the exponential phase of culture compared with cells from the stationary phase of growth (*p <* 0.05) (Table 2).

**Table 2:** Intracellular vitrification temperature for *L*. *bulgaricus* CFL1 following growth under different conditions, following the addition of cryoprotective additive and subsequent wash (Tg’) determined by DSC.

Cryoprotective conditions	Intracellular Tg’ (°C)			
	MRS		WHEY	
	Exponential	Stationary	Exponential	Stationary
Control—Peptone water	-18.8 ± 0.9^b^	-16.2 ± 1.4^a^	-17.6 ± 0.8^ab^	-15.8 ± 1.4^a^
Sucrose–no wash	-27.8 ± 1.8^d^	-26.5 ± 0.7^d^	-28.6 ± 3.1^d^	-27.9 ± 2.4^d^
Sucrose–one wash	-22.2 ± 0.9^c^	-20.0 ± 1.0^c^	-17.1 ± 0.8^ab^	-17.4 ± 0.4^ab^
Glycerol–no wash	-45.1 ± 0.6^f^	-45.8 ± 0.6^f^	-33.0 ± 2.6^e^	-32.7 ± 1.7^e^
Glycerol–one wash	-27.2 ± 1.0^d^	-22.4 ± 1.2^c^	-17.4 ± 2.0^ab^	-14.7 ± 0.8^a^
DMSO–no wash	-51.1 ± 1.9^f^	-50.0 ± 2.5^f^	-51.5 ± 0.8^f^	-49.7 ± 3.4^f^
DMSO–one wash	-33.0 ± 2.9^e^	-33.3 ± 2.6^e^	-19.3 ± 1.1^b^	-17.5 ± 1.0^ab^

Tg’ is the mean of 3–4 independent measurements ± experimental standard deviations. Superscripts letters (a, b, c, d, e, f) represent statistical contrasts (significant differences) between samples at the 95% confidence level.

**Fig 4:**
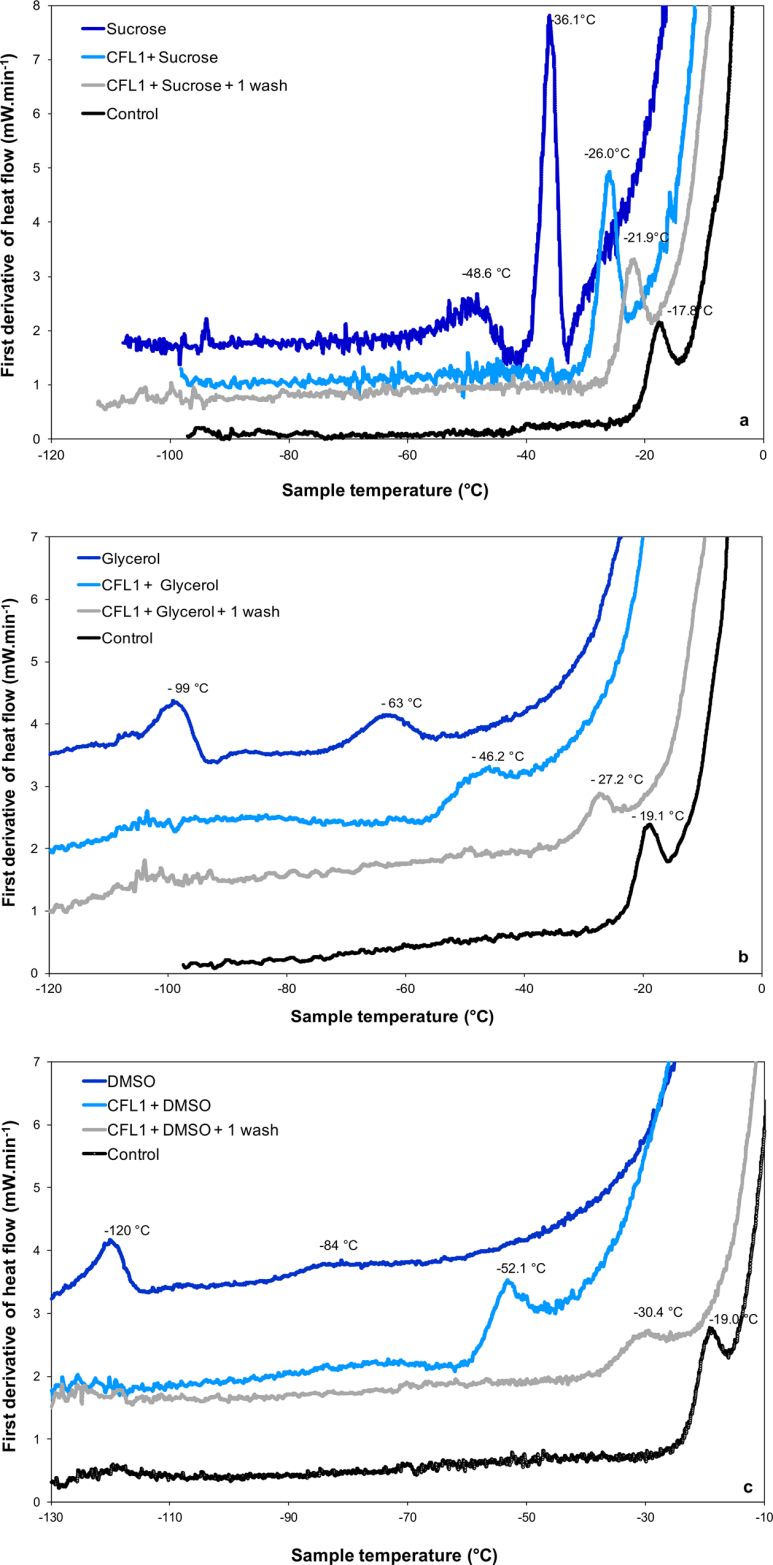
DSC first derivative of heat flow of *L*. *bulgaricus* CFL1 with cryoprotective additives and aqueous solutions of cryoprotectants. (a) sucrose, (b) glycerol and (c) DMSO are all additives at 0.58 M. The vitrification temperatures of incubated (light blue curve) and subsequently washed cells (grey curve) are indicated. Samples were cooled below −90°C and then warmed at 10°C.min^-1^. Control samples (washed with peptone water) (black curves) and aqueous solutions of cryoprotectants (dark blue curves) are also presented for comparison.

The effect of the addition of protectants commonly used in cryopreservation and freeze drying on the intracellular Tg’ of *L*. *bulgaricus* CFL1 was determined by DSC. The effect of the additives: sucrose, glycerol and DMSO on the intracellular transition of exponential phase *L*. *bulgaricus* in MRS growth medium are shown in Fig 4.

In the control cells intracellular Tg’ was observed at -18.8 ± 0.9°C (Table 2). In a cell free system two transitions are observed representing the Tg’ of the maximally freeze concentrated phase (-48°C, -99°C and -120°C, for sucrose, glycerol and DMSO in a 0.15 M NaCl solution, respectively) and the softening temperature at which the system exhibits an observable deformation (viscous flow in real time) under its own weight (-36°C, -63°C, -84°C) respectively (Fig 4, dark blue traces).

Addition of the cryoprotective additive sucrose had a minor effect on intracellular Tg’, reducing the Tg’ of MRS-grown cells by 9°C (exponential phase, Fig 4A) and 10°C (stationary phase) and of whey-grown cells by 11°C (exponential phase) and 12°C (stationary phase) (Table 2). Washing cells to remove the sucrose shifted Tg’ close to the control values, with no differences between the washed cells and control in the case of whey-grown cells (*p <* 0.05).

The addition of glycerol shifted intracellular Tg’ in MRS grown cells by 26°C (exponential phase, Fig 4B) and 30°C (stationary phase). One washing step results in a 28°C increase of the intracellular Tg’ (-27.2°C), bringing it closer to the control value. With whey-grown cells the observed reduction in Tg’ was less than in MRS-grown cells, being 16°C (exponential phase) and 17°C (stationary phase). Washing the cells returned Tg’ close to the control values (Table 2).

With DMSO the intracellular Tg’ was reduced in both MRS- and whey-grown cells by 33°C (Fig 4C), with no significant differences observed between exponential and stationary phases of growth. Washing MRS-grown cells following exposure to DMSO resulted in a 50% change in this DMSO induce shift in Tg’. In whey-grown cells exposed to DMSO and washed, Tg’ values were restored to control values (*p* < 0.05).

**Fig 5:**
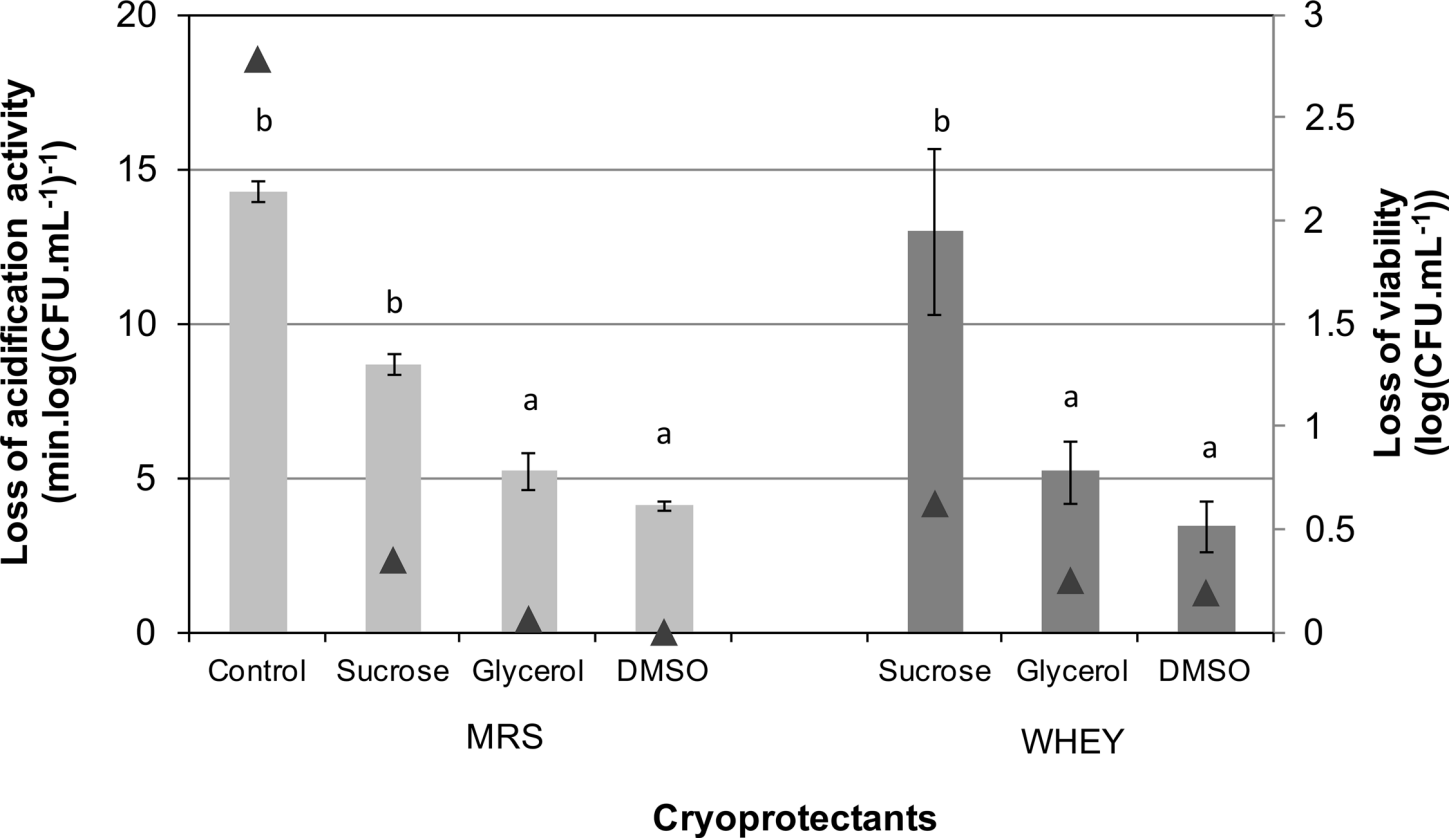
The effects of cryoprotective agents on the loss of specific acidification activity and viability upon freeze-thawing of *L*. *bulgaricus* CFL1. Cells were grown in either whey or MRS medium, harvested in stationary culture phase and frozen to -80°C at 2°C min^-1^ with sucrose, glycerol or DMSO (all additives at 0.58 M) and no additive (control: 1 g.L^-1^ peptone water). Superscripts letters represent statistical contrasts (significant differences) between samples at the 95% confidence level. Control whey grown cells displayed an extreme loss in cell function (64.8 ± 3.5 (min.log(CFU.mL^-1^)^-1^)) and viability upon thawing (3.3 ± 0.3 log(CFU.mL^-1^)), and for clarity are not included in this figure.

To assess the viability and the activity of *L*. *bulgaricus* following freezing and thawing, cells were cooled slowly (2°C.min^-1^) and thawed rapidly. Under these conditions, cells with no cryoprotectant show significant loss of viability (2.7 log(CFU.mL^-1^) for MRS-grown cells and 3.3 log(CFU.mL^-1^) for whey-grown cells) and acidification activity (14.3 min.log(CFU.mL^-1^)^-1^ for MRS-grown cells and 64.8 min.log(CFU.mL^-1^)^-1^ for whey-grown cells), whereas cells with cryoprotectant show significantly enhanced activity on thawing (Fig 5). With both whey-grown and MRS-grown cells the viability and activity on thawing is determined by the cryoprotectant used. With the experimental conditions used, DMSO and glycerol give the highest and similar activity on thawing (*p* < 0.05) followed by sucrose. MRS-grown cells were more resistant to freeze injury than whey-grown cells. Control cells, frozen in the presence of peptone water exhibited as expected the highest losses of viability and acidification activity.

**Fig 6:**
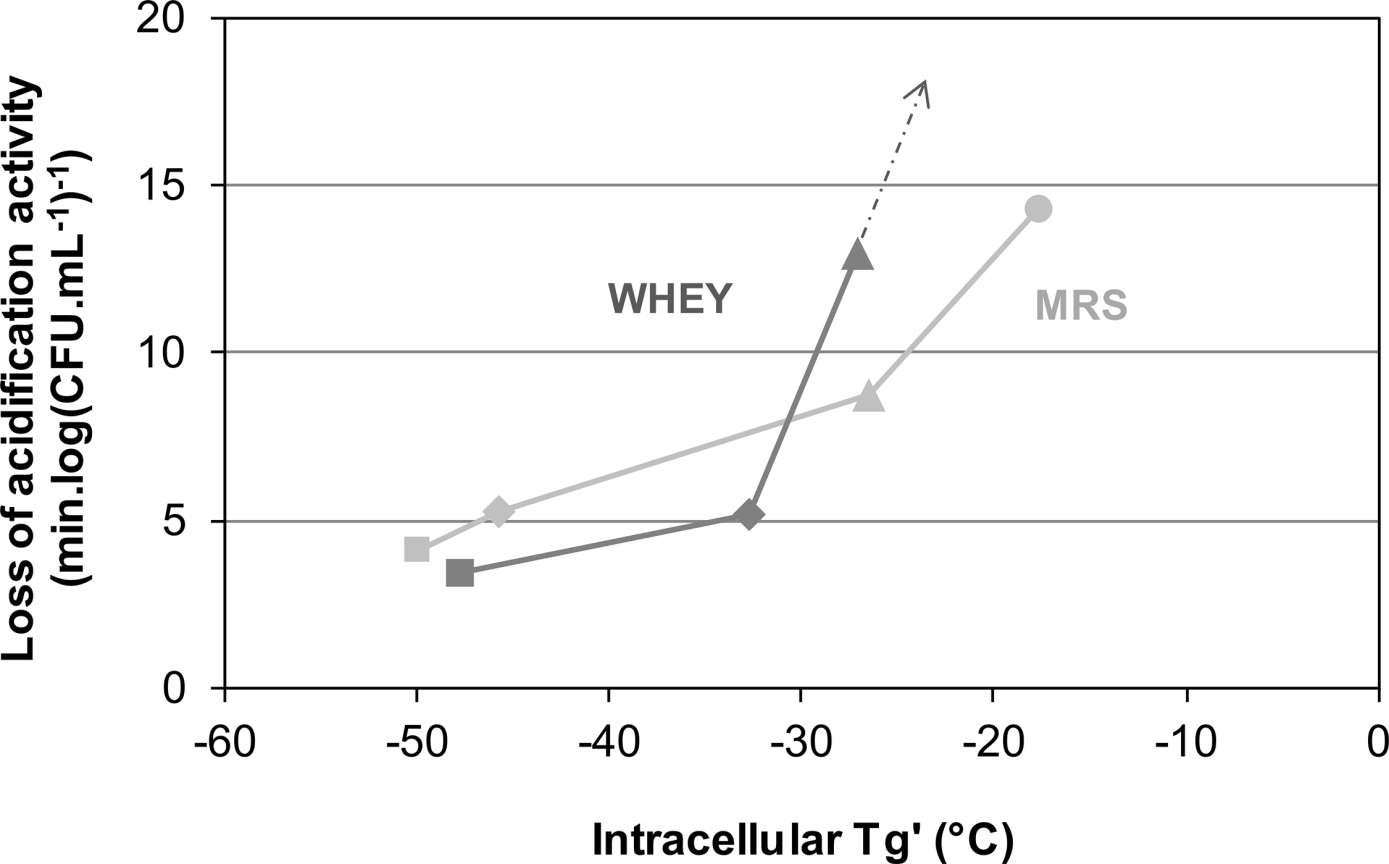
The loss of specific acidification activity on thawing of *L*. *bulgaricus* plotted against intracellular vitrification temperature (Tg’). Cells were grown in either whey (dark grey) or MRS medium (light grey), harvested in stationary culture phase and frozen to -80°C at 2°C min^-1^ with sucrose (triangles), glycerol (diamonds) or DMSO (squares), (all additives at 0.58 M) and MRS control (circle). Dotted arrow indicates the direction of control whey grown cells loss in cell function (64.8 ± 3.5 (min.log(CFU mL^-1^)^-1^)).

A plot of the loss of acidification activity on thawing against intracellular Tg’ (Fig 6) shows that cells with the lowest value of intracellular Tg’ survive the freezing process better than cells with a higher intracellular Tg’.

## Discussion

### Intracellular glass transitions in microorganisms

It is now recognized that the intracellular compartment is an aqueous environment which is extremely crowded. Osmotic removal of water from the cytoplasm leads to a buildup in the concentration of intracellular solutes and an increase in the packing density of macromolecules. In osmotically stressed eukaryotic cells an intracellular glass transition has been reported [6,7]. The intracellular environment of prokaryotes is more packed than eukaryotes [8]. Following osmotic stress in bacteria the macromolecule concentration may exceed 400 g.L^-1^ [8] leading to the formation of a colloidal glass [11] and the diffusion rates for intracellular molecules decrease [9,10].Once this glass has formed, the very high viscosity severely restricts the diffusion of oxygen and metabolites, the cell moves to a dormant state. This switch is driven by changes to the internal state, although our experiments show that these changes are themselves driven by changes in the external environment (as argued in [11]). It has been suggested that anhydrobiotic organisms, including microorganisms, survive desiccation by the formation of intracellular glasses [22].

In a eukaryote, the intracellular compartment is more complex than in a prokaryote such as *Lactobacillus*. Freeze concentration can lead to a colloid glass transition, but there is also the possibility of a glass transition in the intracellular fluid once this has achieved maximal freeze-concentration driven by the formation of intracellular ice.

### Intracellular ice and colloid glass transition in *S*. *cerevisiae*

The eukaryote *S*. *cerevisae* provides a useful model system to validate that the intracellular ice transition can be detected by DSC and that this is distinct to the colloid glass transition. In *S*. *cerevisae* intracellular freezing is detected by DSC as the release of latent heat associated with the change in entropy accompanying the phase transition from liquid water to ice in the cell during cooling, or the reverse during warming. This has been reported previously for rapidly cooled *S*. *cerevisiae* [3] and clearly shown here in *S*. *cerevisiae* cooled at 50°C.min^-1^ (Fig 3A). The intracellular ice signature as detected by DSC (peak B in Fig 2A) correlates with the visualization of intracellular ice by light cryomicroscopy (2] and cryoSEM (Fig 3A)[23]. At rates of cooling of less than 10°C.min^-1^ cells become extensively dehydrated with no evidence of intracellular ice (Fig 3B) [2,3] and no DSC signature of intracellular ice formation is observed during cooling (Fig 2A). Following cooling at 10°C.min^-1^ and 2°C.min^-1^ intracellular vitrification was detected at -17°C during warming by the variation in heat flow associated with the change in heat capacity as described previously [12]. The DSC signal observed at -17°C during warming, is not a membrane lipid transition, as this occurs at higher temperatures (Tm -3.5°C) in *S*. *cerevisae* (S1 Fig). No vitrification signal was seen in control runs (medium without cells). The signal detected in the cell suspension is the glass transition temperature of the maximally freeze-concentrated intracellular compartment (conventionally designated Tg’). A colloid glass transition has been demonstrated in *S*. *cerevisiae* exposed to sorbitol solutions which induce a reduction in cell volume to 40% of the isotonic value [24]. This observed vitrification of the intracellular environment was compatible with cell recovery on removal of the osmotic stress.

Following cooling at 50°C.min^-1^, conditions under which intracellular ice formed, intracellular Tg’ was detected at -17°C during warming. In these cells we assume that the intracellular environment has become maximally dehydrated due to the formation of intracellular ice, leading to the vitrification of the residual unfrozen compartment.

Whatever the cooling rate applied, the Tg’ value shifted to lower temperatures after a second freeze-thawing cycle. Treatments which damage the cellular membranes such as repeated freezing would tend to lead to the loss of small molecules from the vacuoles to the intracellular fluid, thus resulting in a decrease of the temperature of the glass transition. This explanation would be consistent with the value of Tg’ observed resulting from the interaction of proteins and small molecules.

### Intracellular ice and the colloid glass transition in prokaryotes

In *L*. *bulgaricus* and other prokaryotes, intracellular ice has not been detected other than following rapid cooling (> 1000°C.min^-1^) of cells suspended in distilled water [4,25,26]. We did not detect any intracellular ice signature in any of the DSC scans during cooling of *L*. *bulgaricus* in this study or with other prokaryotes [12]. The high intracellular concentration of macromolecules in prokaryotes [8] would indicate that during cooling in the presence of extracellular ice, which would induce osmotic shrinkage, that intracellular vitrification would occur before ice nucleation [12]. A similar argument has been made for the absence of intracellular ice within mammalian spermatozoa [27]. By contrast the eukaryote *S*. *cerevisae*, is predicted to have a lower macromolecule concentration and a larger volume, and during rapid cooling intracellular ice was observed to nucleate at a temperature (-16.7°C) warmer than intracellular Tg’.

At slow cooling, in the absence of any cryoprotective agent, and provided that the cell membrane is still osmotically active, intracellular Tg’ is observed to occur between -10°C and -25°C for a range of prokaryotes [12]. We assume that this glass transition is that of the freeze concentrated proteins within the cell. The DSC signal observed is not a membrane lipid transition, which occurs at +8°C to +10°C in *L*. *bulgaricus* CFL1 during cooling and at +29 to +32°C during heating (S2 Fig and S1 Table). The glass transition temperatures of freeze concentrated protein solutions have been demonstrated to occur between -10°C and -14°C [27–32]. It has been demonstrated that the addition of solutes, such as sugars to protein solutions reduces the glass transition and softening temperatures. For example, the sucrose at a ratio 65/35 BSA/sucrose increased Tg’ to -24°C, while a higher content of sucrose in the formulation (10/90 BSA/sucrose) Tg’ shifted to -33°C [30]. The differences observed in the Tg’ of different species [12] and following different growth conditions (Table 2) may simply reflect different intracellular levels of proteins and small molecules between species or treatments.

The addition of molar amounts of common permeating and non-permeating cryoprotectants to *L*. *bulgaricus* modified intracellular Tg’, with the effects being observed were dependent on the type of cryoprotectant and the growth conditions of the cells (Table 2). The addition of DMSO reduced the measured Tg’ by approximately 32°C for all the experimental variables, with no significant effect of growth medium or stage of culture. Washing cells with DMSO free media caused an increase in intracellular Tg’ close to the control value (no additive). These observations suggest that DMSO is freely permeable to *L*. *bulgaricus*.

The effect of glycerol on Tg’ was dependent on the growth conditions. The Tg’ values in MRS grown cells reduced by at least 25°C following the addition of glycerol, whilst with whey grown cells the observed shift in Tg’ was much less (15°C). Washing with glycerol free medium increased Tg’ to near control values (Fig 4B, Table 2). Glycerol is not considered to be freely permeable to *L*. *bulgaricus*, but there are known glycerol facilitator proteins [33] which actively transport glycerol into the cell, and it is possible that whey grown cells and MRS cells may express different levels of these proteins. The presence of intracellular glycerol will be expected to have similar effects to that of DMSO, the differences observed between the two growth conditions may reflect different internal concentrations of glycerol and intracellular proteins.

Sucrose is not considered to be a permeating additive for *L*. *bulgaricus* but its presence in the extracellular medium reduced the intracellular Tg’ by 10°C. This was unaffected by variations in the growth medium or stage of culture. It is difficult to know how to interpret these results, although they may reflect the interaction between sucrose and the cell envelope. In all cases the values of intracellular Tg’ observed here are intermediate between the values observed for either intracellular proteins or cryoprotectants alone.

The extracellular medium, the aqueous solutions of cryoprotectants alone at the same concentrations (0.58 M), present Tg’ values of -12°C for DMSO, -99°C for glycerol and -48°C for sucrose (Fig 4). They are all lower than the corresponding intracellular Tg’ of bacterial cell after exposure to cryoprotectants (Table 2). In the absence of additives Tg’ is observed at a much higher temperature and the effect of the additives is to fluidise the intracellular environment.

**Fig 7:**
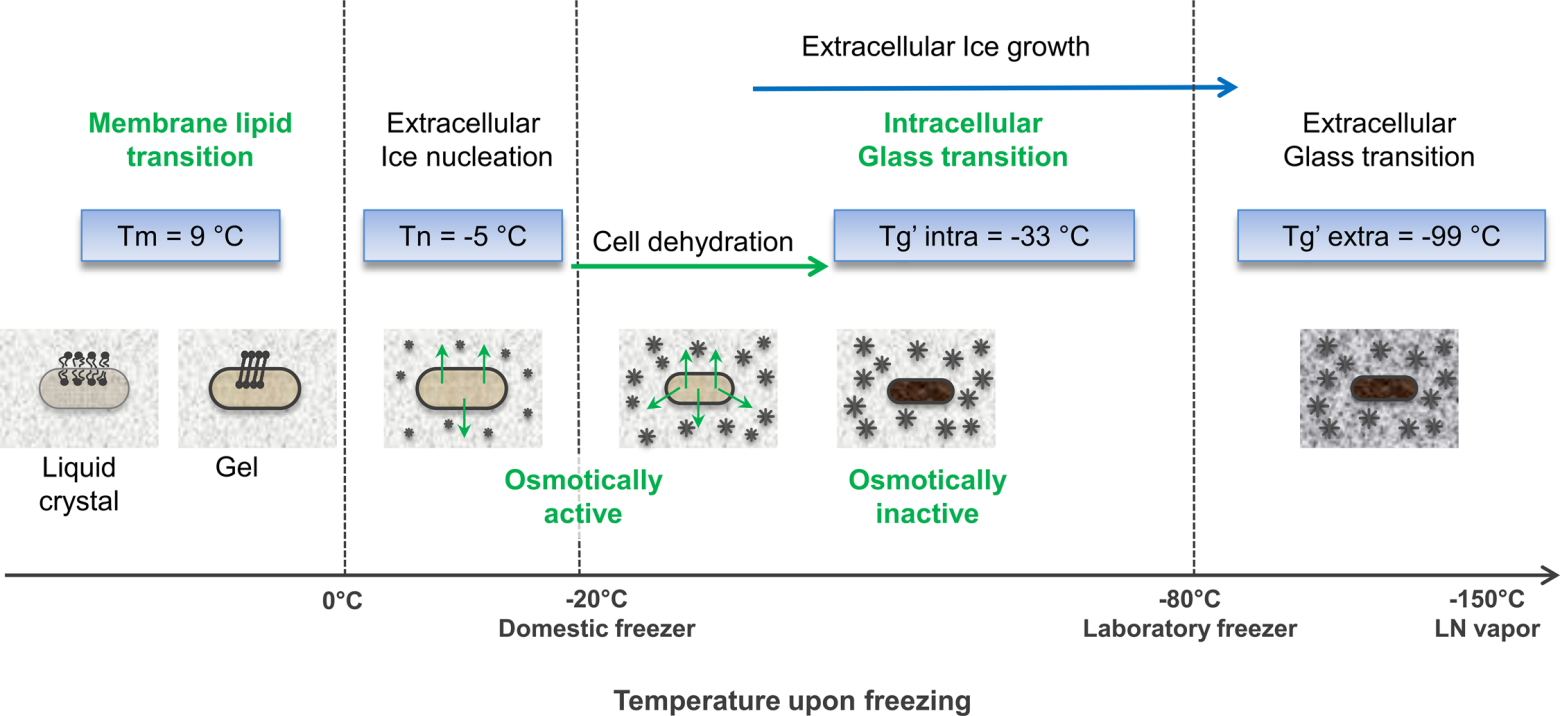
Schematic of the transitions which occur during cryopreservation of *L*. *bulgaricus* CFL1. Temperature transitions correspond to cells grown in whey medium and protected with glycerol (0.58 M).

Our measurements of Tg’ of the intracellular and the extracellular media allow us to define the temperatures of the various transitions that occur in cells and the surrounding media during cooling and warming in different cryoprotectants compared at the same concentration. The membrane phase transitions temperatures for *L*. *bulgaricus* CFL1 grown on MRS and whey are known [17]. In addition we have shown using FTIR that additives (DMSO, sucrose and glycerol) have no major effect on membrane phase behavior in whey grown cells (S2 Fig and S1 Table). By the time ice forms in the extracellular environment the membrane lipids will be in a gel state for all cases examined here. Fig 7 illustrates the physical events taking place during a slow freezing of cells. Cells grown in whey and suspended in glycerol will partition into a freeze concentrated compartment, osmotic shrinkage will increase during cooling and at -32.7°C the partially dehydrated intracellular environment will undergo a colloid glass transition. Following intracellular glass formation the cells will not be osmotically responsive to any further increase in external hypertonicity. The extracellular medium will be very viscous and will undergo a glass transition at -99°C. During warming these processes are reversed and between -99°C and -33°C the vitrified cells are exposed to a fluid extracellular environment.

The effects of DMSO and sucrose on cellular transitions in *L*. *bulgaricus* will qualitatively be similar to that observed in glycerol suspended cells.

### Intracellular Tg and cryopreservation

When sucrose, glycerol and DMSO are compared at an equal concentration, which on a colligative basis would be expected to have a similar effect on the fraction of the unfrozen compartment, the viability on thawing correlates well with the temperature of intracellular vitrification: cells in which intracellular vitrification was delayed had a lower loss of activity and a higher viability on thawing (Fig 6). A low intracellular Tg’ which means that cells are capable of osmotic shrinkage at low temperatures during cooling, appears to be protective compared with those treatments with a high intracellular Tg’ which would become osmotically unresponsive at higher temperatures. Of course these additives have many other effects during freezing and thawing and for example will vary in their viscosity, cell permeability, modification of membrane structure and fluidity etc. factors which could independently modify viability. The molecular basis for the efficacy of additives for any specific cell type and the differences between diverse cell types are not understood. However, the data presented in Fig 6 illustrates the potential effect of the physical state of the intracellular environment on viability and metabolic activity following cryopreservation.

### Storage temperatures

From a practical point of view it is important to understand what determines stability at low temperatures and to develop strategies to allow storage at higher subzero temperatures. During cryopreservation cells become concentrated into regions of freeze concentrated solute and it has been proposed that it is the molecular glass transition temperature of this extracellular material which determines long term viability during storage. At temperatures colder than Tg’ of the extracellular compartment, no diffusion can occur and cell viability is expected to be independent of storage time. At temperatures warmer than Tg’ of the extracellular compartment diffusion can occur, leading to chemical reactions at interface of the cell with its surrounding environment and cell viability is expected to decline with time. The rate of loss of viability is determined by a non-linear deviation from Tg’. From this study it is also apparent that the intracellular environment forms a glass at a higher temperature than the extracellular environment and further studies should be carried out to determine the relative importance of intra- and extra-cellular Tg’ in determining viability at different storage temperatures.

## Conclusions

The data presented here allows discussion of the role of the physical state of the intracellular environment in determining the response of microbial cells to preservation and could be a powerful tool to be manipulated to allow the optimization of methods for the preservation of microorganisms.

## Supporting Information

S1 Fig Membrane phase behavior of *Saccharomyces cerevisiae* 338 cells upon cooling and warming. Position of the νCH_2_ versus temperature plots (thin lines) upon cooling (in blue) and warming (in red), and the corresponding inverted second derivatives (thick lines) are presented. The membrane lipid transition temperatures correspond to the maximum of the second derivatives and are indicated by the dashed lines.
(TIF)

S2 Fig Membrane phase behavior of *Lactobacillus delbrueckii* ssp. *bulgaricus* CFL1 cells grown in whey broth upon cooling and warming. Position of the νCH_2_ versus temperature plots (thin lines) upon cooling (in blue) and warming (in red), and the corresponding inverted second derivatives (thick lines) with sucrose, glycerol, DMSO, as additives or no additive are presented. The membrane lipid transition temperatures correspond to the maximum of the second derivatives and are indicated by the dashed lines.
(TIF)

S1 Table Summary of the lipid membrane transition temperatures of *L*. *bulgaricus* CFL1. Cells were grown in whey broth, with additives (0.58 M) or no additive, upon cooling to -50°C and heating to +80°C.
(TIF)
