# Supplementary figures and images for: Determination of Intracellular Vitrification Temperatures for Unicellular Micro Organisms under Conditions Relevant for Cryopreservation
Source: PLoS One. 2016 Apr 7;11(4):e0152939. doi: 10.1371/journal.pone.0152939 (PMC4824440; doi:10.1371/journal.pone.0152939)

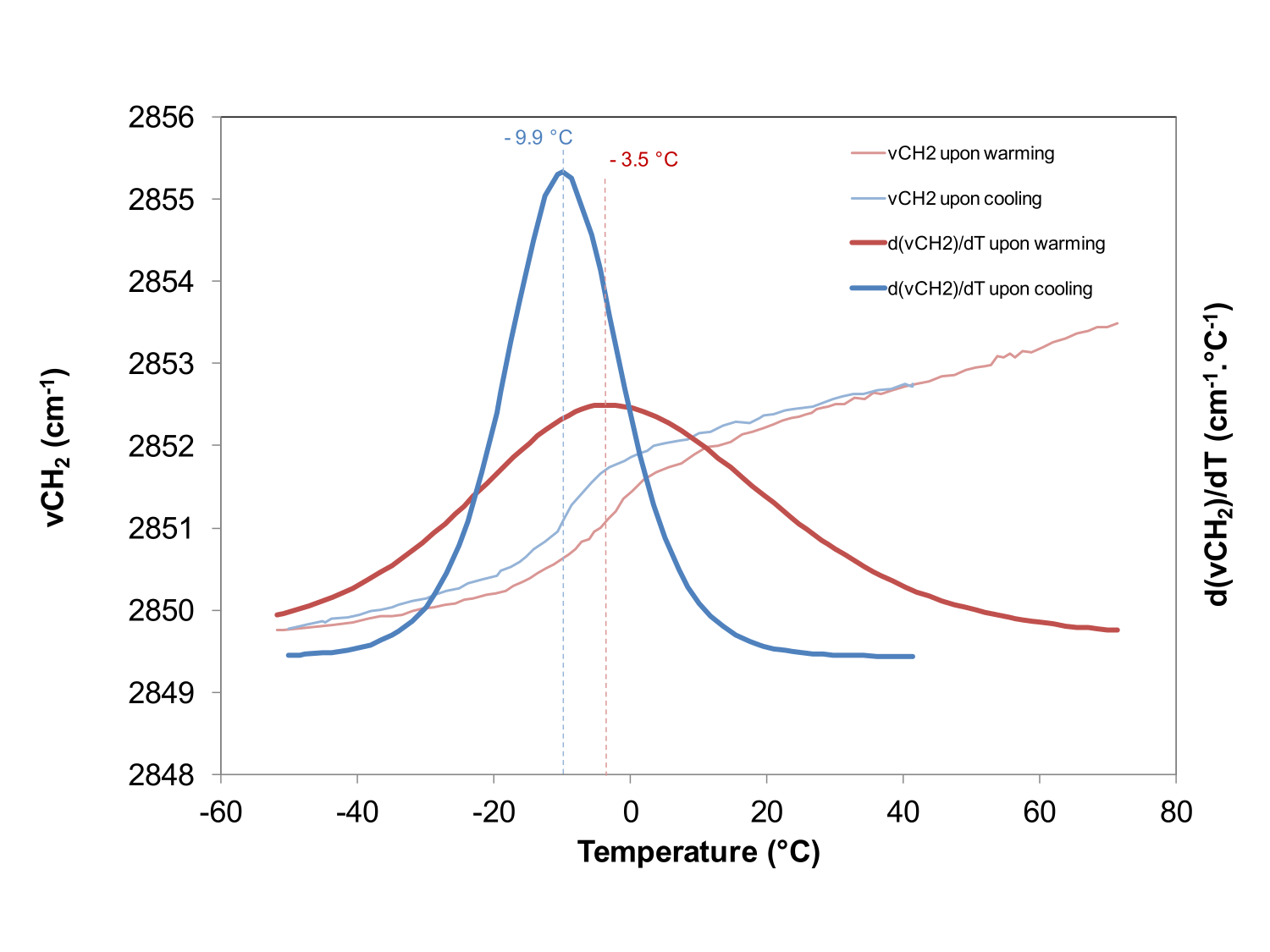

Supplement: S1 Fig — Position of the νCH2 versus temperature plots (thin lines) upon cooling (in blue) and warming (in red), and the corresponding inverted second derivatives (thick lines) are presented. The membrane lipid transition temperatures correspond to the maximum of the second derivatives and are indicated by the dashed lines. (TIF) [file pone.0152939.s001.tif]

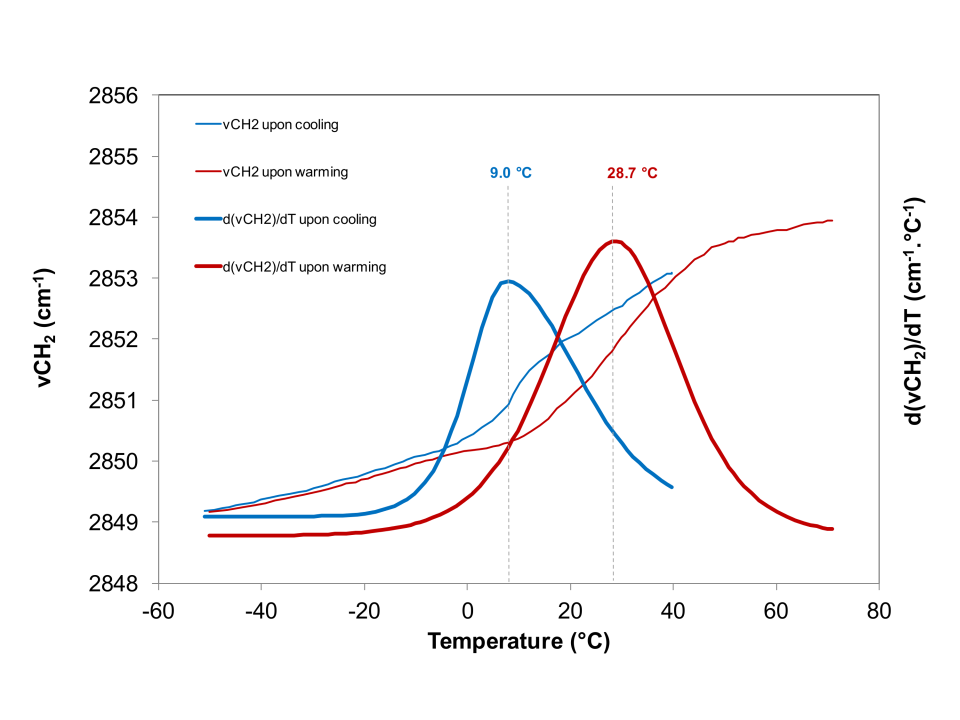

Supplement: S2 Fig — Position of the νCH2 versus temperature plots (thin lines) upon cooling (in blue) and warming (in red), and the corresponding inverted second derivatives (thick lines) with sucrose, glycerol, DMSO, as additives or no additive are presented. The membrane lipid transition temperatures correspond to the maximum of the second derivatives and are indicated by the dashed lines. (TIF) [file pone.0152939.s002.tif]

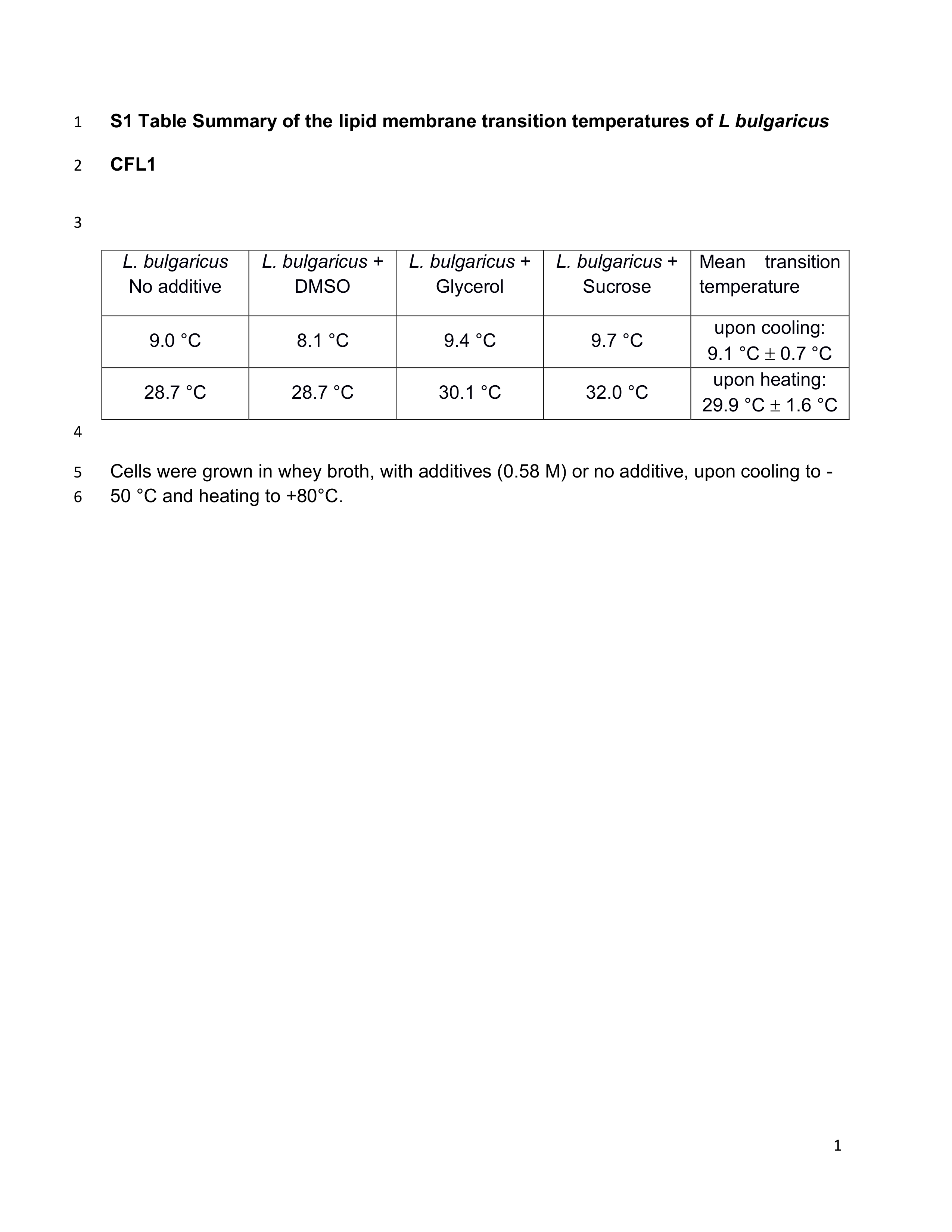

Supplement: S1 Table — Cells were grown in whey broth, with additives (0.58 M) or no additive, upon cooling to -50°C and heating to +80°C. (TIF) [file pone.0152939.s003.tif]
